# Characteristics of People Returned to Prison From Medium Secure Psychiatric Services in England and Wales: National Cohort Study

**DOI:** 10.3389/fpsyt.2022.881279

**Published:** 2022-06-29

**Authors:** Sarah Leonard, Roger T. Webb, Michael Doyle, Jennifer Shaw

**Affiliations:** ^1^Centre for Mental Health and Safety, University of Manchester, Manchester, United Kingdom; ^2^Department of Nursing and Midwifery, University of Huddersfield, Huddersfield, United Kingdom

**Keywords:** forensic mental health services, secure services, violence risk assessment, prison transfer, prison

## Abstract

**Background:**

Little is known about people who are admitted to medium secure services (MSSs) from prison, including characteristics and factors that influence clinical pathways and subsequent discharge. We recently published the first study to establish the circumstances by which MSS “prison-transfer” patients are returned to prison. Of particular concern was the finding that a quarter of prison-transfer patients were returned to prison by Responsible Medical Officers (RMOs) because they were not engaging with treatment or were deemed too “high risk” to remain detained within the services, circumstances that would be unacceptable when considering discharge via a community care pathway. It is important to further explore the characteristics of people admitted to MSSs from prison, and to investigate how these may differ for individuals who are returned to prison, as compared to those discharged into the community.

**Aim:**

(a) To describe the characteristics of prison-transfers who receive an RMO directed discharge from MSSs; and (b) to compare these characteristics by discharge destination; prison return and community discharge.

**Methods:**

Prospective cohort comparative study: all prison-transfer patients discharged under the instruction of their RMO over a 6-month period, from 33 NHS medium secure units across England and Wales. Data on patient demographic, clinical and legal characteristics were extracted via full patient health record review and collateral information from clinicians was also obtained. This information was used to complete The Historical, Clinical and Risk-−20 items (HCR-20v3) and The Structured Assessment of Protective Factors (SAPROF). Individuals who were returned to prison were compared with those who were discharged to the community.

**Results:**

Persons returned to prison represented a vulnerable group at time of discharge as compared to those discharged into the community and had a significantly shorter length of stay in MSSs. Over half of those returned to prison had a length of stay of <6 months. Individuals returned to prison displayed significantly more issues with psychological adjustment at time of discharge, and had a higher risk of future violence and a lower prevalence of protective factors that mitigate subsequent risks of relapse and reoffending.

**Discussion:**

MSs in England and Wales are returning vulnerable individuals to prison in lieu of adequate aftercare services. The role of and responsibilities of MSSs as regards admissions from prison needs to be reconsidered.

## Introduction

The prevalence of psychiatric disorders within the prison population in England and Wales is high (1, 2). Figures from NHS England suggest that 10% of prisoners are in treatment for mental illness, although it is recognised that there may be more people in treatment who are not captured in these data (3). This is higher than figures for the general population, of which 5% are estimated to access secondary mental health services (4). For individuals in prison in need of psychiatric in-patient care, treatment and therapeutic intervention is provided via transfer to secure psychiatric services (5). This pathway is for patients for whom appropriate care cannot be given in a prison environment and those deemed to require compulsory treatment. Transfer to secure psychiatric services can also be directed by the criminal courts for assessment to inform sentencing decisions, or courts may impose a hospital treatment order in place of/alongside a custodial sentence [see Supplementary Table 1 for part 3 of the Mental Health Act 1983 (MHA)]. These services operate at three levels (low, medium, and high), providing a range of physical, procedural and relational security measures to ensure effective treatment. Progress and transition through secure psychiatric services should be determined by the individual's level of engagement with their treatment/care plan, evidence of reduction in risk of harm to others and reduced need for care and supervision (6–8). Following treatment completion patients are discharged to a destination such as the community, another in-patient service or are returned to prison. These decisions are managed by the patients' Responsible Medical Officer (RMO), and discharge options available to RMOs are often constrained by the patients MHA section and legal status; for instance, if the patient is sentenced or on remand/awaiting sentencing. Criminal courts may also direct the discharge of a patient via court process; for example, imposing a custodial sentence. This can take place with or without the support of the RMO. Transition from secure psychiatric services is a vulnerable period in a patient's care pathway, one that is observed to be associated with increased risk of relapse, reoffending, and suicide and other causes of death for individuals discharged into the community (9, 10). There exists a large body of research investigating patients who are transitioned from secure services into the community (9). In contrast, little research has been conducted internationally on patients who are returned to prison following treatment.

More than one in five patients are now discharged from medium secure services (MSS) back to prison in England and Wales, and elevated risk has been observed in this population as compared to those discharged into the community (11). We recently published the first study to establish the circumstances by which “prison-transfer” patients in secure psychiatric service are returned to prison (12). Over a 6 month period we documented all remittals to prison from 33 National Health Service (NHS) MSS. Of the 96 people remitted, it was observed that 16 (17%) were returned to prison following a court process; receipt of a custodial sentence (*n* = 12), re-remanded to await trial (*n* = 4). Fourteen were with the support of the patients RMO and two were not. The remaining 80 (83%) patients were remitted under the instruction of their RMO. Forty-four were remitted owing to treatment completion, to continue their custodial sentence or await trial, and nine were remitted owing to the responsible clinician not detecting evidence of symptomatology that would warrant detention in a MSS. Of particular concern was the finding that 25 (26%) were remitted owing to non-engagement (*n* = 15) or owing to presenting as too “high risk” to continue to be detained in MSSs (*n* = 10). Return to prison on these grounds was unexpected, given that discharge of non-engaging or high-risk patients into the community from MSS would be unacceptable (11). It was also observed that 17% of those returned to prison were documented as eligible for parole and/or close to their earliest release date at the time of return. It was unclear why these patients did not remain in MSS until the end of their custodial sentences, to maximize the likelihood of successful transition into the community. It is difficult to insert these findings into an international context as return to prison is a widely undocumented clinical practice within the forensic mental health literature. It is not possible to view these findings within the context of the international literature as remittal to prison is a widely undocumented clinical practice within the forensic mental health literature. Since the publication of this work, however, remittal circumstances over a 10 year period have been extracted from a MSS in Belgium (13). It was observed that all remitted patients were returned on the basis that they subverted service regulations. This included: threats of violence; physical violence; instigation of a riot; criminal recidivism; drug trafficking, and bullying and harassment. A further study observed that remitted patients had significantly a significantly higher instance of traits of psychopathy and substance use problems, alongside significantly more static and dynamic risk factors for violence at time of remittal as compared to those who remained detained in the MSS (14). These findings suggest that remittal of patients who are “high risk” is a clinical practice that is not unique to England and Wales.

We also conducted a subsequent qualitative investigation with clinicians working in forensic psychiatric services to understand discharge decision-making for individuals admitted from and returned to prison (15). Clinicians shared the context of constraints in which they operate and the ways in which they perceive these constraints. However, most relevant to the current investigation were the discretionary pathway decisions that RMOs make when prioritizing patients for continued in-patient treatment, and directing remittal for others. Across MSS-based clinicians, there was a clear drive to protect the remit of their service, as they viewed it within the context of the wider forensic mental health system. Admission from prison and prolonged length of stay was described as a “valuable opportunity”, and characteristics of “appropriate” patients were proposed. The degree to which treatment non-engagement and high-risk behaviors were tolerated was described as being dependent on an individual's primary diagnosis, whereby those with a personality disorder were more likely to be remitted to prison on this basis. Prison-based clinicians, however, described how, in these circumstances, prison was being judged inappropriately as a “safe” discharge destination, and expressed concern about the limited services available for these patients after their return to prison, and the lack of resources to prepare for their subsequent release from custody into the community. Indeed, in our previous study we found that <20% of patients returned to prison who had a legal entitlement to section 117 aftercare under the MHA were receiving care managed/delivered via the Care Programme Approach (a person-centered care plan coordinated by a named clinician). Subsequent pathways for remitted patients included: inter-prison transfer (30%), use of the Assessment, Care in Custody and Teamwork (ACCT) process (49%, a care plan system for people at elevated risk of suicide or self-harm), re-referral to secure services (21%) and community release (30%), with less than half of community releases receiving a referral to community mental health team (10).

Findings from both studies indicate that individuals who are remitted to prison are a vulnerable group of patients, many of whom require interventions such as advanced monitoring in the absence of targeted aftercare services. At present, more information is required about the characteristics and clinical presentation of prison-transfer patients who are returned to prison, to provide insight into their clinical needs prior to their return. It is clear that criteria considered for those being returned to prison is different from criteria for those discharged via a community care pathway. As such, comparison of characteristics and clinical presentation at time of remittal/discharge into the community will provide insight into the thresholds applied to prison-transfer patients who are transitioned via each pathway. Suitability for transition from MSSs should be supported by an assessment of the individual's risk and proactive risk management strategies to ensure recovery and rehabilitation. Within England and Wales, this process is supported by evidence-based structured professional judgement tools, most commonly the Historical, Clinical & Risk: 20 items, version 3 (16, 17). In recent years, the use of assessment frameworks which focus on protective factors (individual attributes which mitigate or eliminate risk) are being used alongside well established risk focused assessments to provide a well-balanced and thorough assessment procedure. In this context, we opted to assess individual characteristics and presentation at time of discharge using the Historical, Clinical & Risk: 20 items, version 3 and The Structured Assessment of Protective Factors for Violence Risk [SAPROF: (18)]. Use of these tools in conjunction represents advancement in assessment of risk, for which there is a growing evidence base (19). Given the circumstances by which the individuals in our study were remitted to prison, it was anticipated that those returned to prison would be assessed as higher risk and to posses fewer protective factors at time of discharge, as compared those discharged into the community.

## Method

### Ethical Approval

An application was submitted and accepted by the Confidential Advisory Group, on behalf of the Secretary of State for Health, to conduct the study as a Confidential Inquiry under Section 251 of the NHS Act 2006. This allowed for the processing of NHS patient identifiable information without the individual's consent. All procedures involving human subjects/patients were approved by the North West of England Multi-site Research Ethics Committee (09/H1016/126).

### Cohort Members and Research Sites

Individuals in this national prospective cohort study were all patients discharged from all 33 NHS MSS's in England & Wales over a 6-month period. Eligible patients were those who were;

Discharged into the community or remitted to prison.

Discharged under the instruction of their RMO or with the RMO's agreement (i.e., the RMO had instructed the criminal court process).

Originally admitted/transferred to MSSs directly from a prison establishment for assessment/treatment; i.e., emergency transfers of both remand (s. 48/49 MHA) and sentenced prisoners (s.47/49 MHA). Patients subject to court orders and admitted to MSSs directly from a prison establishment were also eligible for inclusion (s. 38 and 45A MHA) (see Supplementary Table for Mental Health Act sections for patients concerned in criminal proceedings or under sentence).

There were 141 eligible patients. Approvals were gained and electronic and/or hard copies of medical notes were obtained for all patients.

### Measures

A data collection proforma was developed to capture the data extracted from participants' medical records. This proforma allowed for efficient capture of a range of variables identified by the literature as important for understanding and describing the characteristics of patients discharged from medium security units, and was structured using the following 3 distinct subsections (see Supplementary Table 1 for a detailed breakdown): (1) Demographics and legal status; (2) Clinical characteristics and length of stay, and; (3) Violence risk assessment tools prior to discharge.

#### Historical, Clinical and Risk: 20 Items, Version 3

The HCR-20v3 (16) is a structured violence risk assessment instrument. There are 20 items that align risk markers into “past”, “present”, and “future” and incorporate Historical (10 items), Clinical (5 items) and Risk Management (5 items) factors that have been found to be predictive of future violence. The Historical items are static variables whereas the Clinical and Risk items represent current and future dynamic risk. Items are rated on a three-point scale; 0 = “not present”, 1 = “partially present”, and 2 = “clearly present”. Total scores range from 0 to 40.

#### The Structured Assessment of Protective Factors for Violence Risk

The SAPROF (18) is a guideline developed to measure protective factors for violence risk. It is designed to be used in conjunction with a reliable and valid risk assessment tool according to the Structured Professional Judgement method, such as the HCR-20 and related tools. There are 17 items covering Internal (5 items), Motivational (7 items) and External (5 items) factors that have been found to protect against violence. Each item is rated on a 3-pont scale; 0 = “absent”, 1 = “present to some extent” or 2 = “clearly present”. Total scores range between 0 and 34.

Interrater reliability for both measures is presented in Supplementary Table 2.

##### Omitted Items

For the purpose of this study three items from the SAPROF were removed: “Intelligence” (Internal item 1); “Empathy” (Internal item 3), and; “Financial management” (Motivational item 8). Intelligence was removed due to the formal assessment required to rate this item (the SAFROF manual instructs that this item should be omitted if the assessor does not have access to a patient's Intelligence Quotient assessment; information that is not collected for most patients in secure psychiatric services). Financial management was removed due to limited relevance of this item in secure settings, where access to money is restricted and monitored. “Empathy” (I item 3) was also removed as in many cases collateral informants did not feel able to comment due to not conducting offense-related work with the participant in the period prior to discharge, alongside information not being available in participants' case files. For this study participants could receive a total score of 6 (usually 10) on the Internal Subscale and a total of 12 (usually 14) for the Motivational Subscale. Therefore, participants could receive a total score of 28 (usually 34) across all SAPROF items.

A duplicate proforma was also developed which comprised the Clinical and Risk Management to be used in telephone interviews with collateral informants.

### Procedure

The study was conducted concurrently across the 33 medium secure units, with each unit providing notification of planned and actual remittals/community discharges on a fortnightly basis.

Electronic and/or hard copies of Medical records of each patient were accessed by the research team via the NHS healthcare provider and relevant data were extracted to populate the proforma and the Historical subscale of the HCR-20v3. The prospective nature of the study enabled cross-checking of missing data or discrepancies with administrators and clinicians. Types of documents accessed for the purpose of data extraction included: daily nursing and clinical staff records of patient observations, admission assessments, psychiatric and psychological assessments and reports, and discharge summaries.

The dynamic subscales (HCR-20v3 clinical and risk scales) and the SAPROF were rated subject to a thorough evaluation of the patient's presentation preceding discharge from medium secure services. Information used to do this included medical records and collateral information from clinicians who worked with each patient during that time (e.g., named nurses and RMOs). Collateral informants were consulted via telephone call. Staff members were asked questions regarding the patient's presentation during the 6 months prior to discharge and the measures were scored based on the details they provided. Responses from collateral interviews and information extracted from medical records were combined to populate a final data collection proforma for each patient.

### Data Analysis

Data were analyzed using Statistical Package for the Social Sciences (SPSS) for Windows version 22 (IBM Corp, 2013). Frequencies were calculated to describe the characteristics of the sample (demographics and legal status). To identify any significant differences in clinical characteristics and presentation at time of discharge across the two patient groups, the Mann-Whitney U Test was used for continuous variables and the Chi-squared Statistic used for dichotomous variables, across participant characteristics and risk assessment tool scores. Effect sizes are reported for Mann Whitney tests and relative risk for Chi-squared analyses. (*Note*: continuous variables were tested for normality using the Shapiro-Wilk test of normality across community discharges and prison remissions. Deviations from normality were observed for all study variables including violence risk assessment tool totals and subtotals and non-paramedic Mann-Whitney U tests were therefore conducted to assess median difference between community discharges and prison remissions across continuous variables).

## Results

### Demographics and Legal Status

A total of 243 prisoner-patients were discharged from medium secure services between the 6-month recruitment period. Of these, 153 were prison-transfer patients, 141 were eligible for inclusion. Forty-nine (35%) were discharged into the community and 92 (65%) were remitted to prison. The majority of the patients were male (*n* = 132, 94%). Over half of the patients were of White ethnicity (*n* = 87, 62%), 18% (*n* = 25) were Black or Black British, 10% (*n* =14) were Asian or Asian British, and 5% (*n* = 7) were of mixed ethnicity. Median age at admission was 32 years (Inter Quartile Range (IQR = 17) and baseline was 33 years (IQR = 17). Over half of the sample were sentenced prisoners at time of discharge, (*n* = 80, 57%). Just under 20% of patients were subject to an Indeterminate Sentence for Public Protection (*n* = 25, 18%) (see Table 1).

**Table 1:** Demographics and legal status across discharge destination (*n* = 141).

	**All eligible discharges**	**Community discharge**	**Prison remission**
	(*n* = 141)	(*n* = 49)	(*n* = 92)
**Gender**			
Male	132 (94)	42 (86)	90 (98)
Female	9 (6)	7 (14)	2 (2)
**Ethnicity**			
White	87 (62)	28 (57)	59 (64)
Mixed	7 (5)	2 (4)	5 (5)
Asian or Asian British	14 (10)	4 (8)	10 (11)
Black or Black British	25 (18)	12 (25)	13 (14)
Other	8 (6)	3 (6)	5 (5)
**Legal status**			
Sentenced	80 (57)	14 (29)	66 (72)
remand	61 (43)	35 (71)	26 (28)
IPP	25 (18)	3 (6)	22 (24)

*IPP, Indeterminate sentence for Public Protection*.

### Clinical Characteristics and Length of Stay

At 224 days (IQR = 426), median length of stay was below the recommended stay of 18 months to 2 years (14) and length of stay for prison remittals was significantly shorter (median = 173.5 days, IQR = 367) than for community discharges (Median = 404.0 days, IQR = 830) *u* = 1,423.0, *p* < 0.001 (Table 2). Most patients stayed in medium secure services <2 years (*n* = 116, 82%), although those remitted to prison were 66% less likely to be have been admitted for “24 months plus” (10 vs. 33%, *P* =.001). Just under half of patients stayed in medium secure services for 6 months or less (*n* = 61, 43%), and those remitted to prison were twice as likely to be admitted for 6 months or less (53 vs. 25%, p = 0.001).

**Table 2:** Mann Whitney U comparisons of length of stay across discharge destination (*n* = 141).

	**Median (IQR), min. to max. range**						
	**Total**	**Community discharge**	**Prison remission**	***U***	***Z***	***p***	***r***
Length of stay	224 (426) 17–1,912	404 (830) 21–1,912	173 (367) 17–1,822	1,423.00	−3.559	<0.001	0.30

Patients' primary diagnoses were coded as eight distinct categories. The most common diagnosis within the cohort was schizophrenia (*n* = 41, 29%), followed by personality disorder (*n* = 39, 28%) and “psychosis other” (*n* = 19, 14%; including; transient psychotic episode, drug induced psychosis, and first episode psychosis). Schizoaffective disorder, bipolar disorder and “other diagnosis” (including; obsessive compulsive disorder, anxiety disorders, and acquired brain injury) each represented 6% (*n* = 9) of the cohort. Patients with a diagnosis of depression represented 6% (*n* = 8) of the cohort and 5% (*n* = 7) had no psychiatric diagnosis at baseline (due to lack of evidence of illness during patient admission and assessment). Patients who were remitted to prison were 4.7 times more likely to have a primary diagnosis of personality disorder (38 vs. 8%, *p* < 0.001) yet in contrast were 48% less likely to have a primary diagnosis of Schizophrenia (23 vs. 41%, *p* =.025) and 69% less likely to have a primary diagnosis of “psychosis other” (8 vs. 25%, *p* =.005) than those discharged into the community. There was no association between the other 4 primary diagnosis categories and discharge destination likelihood (see Table 3).

**Table 3:** Rate ratio comparisons of clinical characterizes across discharge destination (*n* = 141).

	***n*** **(%)**					
	**Total**	**Community discharge**	**Prison remission**	**χ^2^**	***P***	**Rate Ratio (95% CI)**
	**(*n* = 141)**	**(*n* = 49)**	**(*n* = 92)**			
**Length of stay (2 categories)**						
0–24 months	116 (82)	33 (67)	83 (90)	11.46	0.001	1.34 (1.09, 1.65)
24 months plus	25 (18)	16 (33)	9 (10)			
**Length of stay (5 categories)**						
0–6 months	61 (43)	12 (25)	49 (53)	10.78	0.001	2.18 (0.13, 0.45)
6–12 months	32 (23)	13 (21)	19 (21)	0.63	0.427	0.78 (0.42, 1.44)
12–18 months	15 (11)	5 (10)	10 (11)	0.02	0.903	1.07 (0.37, 2.94)
18–24 months	8 (6)	3 (6)	5 (5)	0.03^*^	1.00	0.32 (0.08, 1.28)
24 months plus	25 (18)	16 (33)	9 (10)	11.46	0.001	0.23 (0.14, 0.63)
**Primary diagnosis**						
Schizophrenia	41 (29)	20 (41)	21 (23)	5.02	0.025	0.52 (0.34, 0.93)
Personality disorder	39 (28)	4 (8)	35 (38)	14.27	<0.001	4.66 (1.76, 12.35)
Schizo-affective disorder	9 (6)	5 (10)	4 (4)	1.74	0.276^*^	0.43 (0.12, 1.51)
Bipolar disorder	9 (6)	4 (8)	5 (5)	0.39	0.719^*^	0.67 (0.19, 2.37)
Psychosis other^a^	19 (14)	12 (25)	7 (8)	7.81	0.005	0.31 (0.13, 0.74)
Other^b^	9 (6)	4 (8)	5 (5)	0.39	0.719^*^	0.67 (0.19, 2.37)
No primary diagnosis	7 (5)	-	7 (8)	3.92	0.096^*^	-
Depression	8 (6)	-	8 (9)	4.52	0.051^*^	-
History self-harm	87 (62)	23 (47)	64 (70)	6.93	0.008	1.48 (1.07, 2.06)
Self-harm prior to admission	52 (37)	8 (16)	44 (48)	13.12	<0.001	2.93 (1.50, 5.72)
Previous MSS admission	27 (19)	5 (10)	22 (24)	3.88	0.049	2.34 (0.95, 5.80)
2nd or sub MSS admission	13 (9)	-	13 (14)	7.63	0.004^*^	-

*2^nd^ or sub MSS admission = second or subsequent admissions to medium secure services during current sentence*.

MSS, medium secure services.

a *Including: “Transient Psychotic Episode”, “Drug Induced Psychosis”, and “First Episode Psychosis”*.

b *Including: “Obsessive Compulsive Disorder”, Anxiety” and “Acquired Brain Injury”*.

* *Fishers exact test used as 25% of expected count <5, and/or observed counts <1*.

For 13 patients (9%), their current medium security admission was their second or subsequent admission to MSSs during their current prison detention. All of these patients were remitted to prison (14 vs. 0%, *p* = 0.004). Likewise, those returned to prison were two times more likely to have had a previous admission to MSSs at some point prior to their current admission (including prior to their current prison detention) (24 vs. 10%, *p* = 0.049). History of self-harming behavior was documented for 62% of the cohort (*n* = 87); this was 1.5 times more likely for prison remittals than for community discharges (70 vs. 47%, *p* = 0.008). For 52 (37%) patients, self-harming behavior was documented to be taking place in prison prior to transfer to MSSs; this was three times more likely for those remitted to prison (48 vs. 16%, *p* < 0.001).

Participants were compared across discharge destination for their total and subscale scores on the validated violence risk assessment tools.

### Historical Clinical-Risk Management 20, Version 3

A Mann-Whitney test revealed that HCR-20v3 total assessment scores were greater for prison remittals (median = 27, IQR = 11.7) than for community discharges (median = 21, IQR = 11.5), *u* = 1,398.50, *p* < 0.001. Across the HCR-20v3 Clinical and Risk Subscales, scores were greater for prison remittals (Clinical, median = 5, IQR = 4; Risk, median = 6, IQR = 3.7) than for community discharges (Clinical, median = 2, IQR = 4, *u* = 1,131.0, <0.001; Risk, median = 4, IQR = 4, *u* = 1,483.0, *p* = 0.001). However, there was no difference between Historical Subscale scores across discharge destination (see Table 4), indicating that the participants had similar histories in regards to previous psychosocial adjustment. See Supplementary Table 3 for a breakdown of individual item presence across the two discharge groups.

**Table 4:** Mann Whitney U comparisons of HCR-20v3 and SAPROF scores across discharge destination (*n* = 141).

	**Median (IQR), min. to max. range**						
	**Total**	**Community discharge**	**Prison remission**	***U***	***Z***	***p***	***r***
**Historical, clinical and risk-−20 items (version 3)**							
Historical factors	15 (6) 4–20	14.44 (5) 4–20	15 (6.4) 6–20	1,864.50	−1.691	0.091	−0.14
Clinical factors	4 (5) 0–10	2 (4) 0–10	5 (4) 0–9	1,131.00	−4.893	<0.001	−0.41
Risk factors	6 (4) 1–10	4 (4) 1–10	6 (3.7) 2–10	1,483.00	−3.365	0.001	−0.31
Total	24 (12) 7–38	21 (11.5) 7–35	27 (11.7) 10–38	1,398.50	−3.706	<0.001	−0.31
**Structured assessment of protective factors**							
Internal factors	3 (2.7) 0–6	4 (2) 0–6	3 (2) 0–6	1,269.50	−2.749	0.006	−0.23
Motivational factors	5 (5.8) 5–22	8 (6.1) 1–12	4.8 (5) 0–11	1,137.50	−4.846	<0.001	−0.41
External factors	6 (1) 2–9	5 (3) 2–9	6 (1) 2–8	1,447.50	−3.589	<0.001	−30
Total	14 (9) 4–25	15.5 (9.2) 4–25	13 (7.8) 5–24	1,492.50	−3.300	0.001	−0.28

### Structured Assessment of Protective Factors for Violence Risk

A Mann-Whitney test revealed that SAPROF total assessment scores were lower for prison remittals (median = 13, IQR = 7.8) than for community discharges (median = 15.5, IQR = 9.2), *u* = 1,492.50, *p* =0.001. Across the SAPROF Internal Factors and Motivational Factor subscale, scores were lower for prison remittals (Internal, median = 3, IQR = 2; Motivational, median = 4.8, IQR = 5) than for community discharges (Internal, median = 4, IQR = 2; Motivational, median = 8, IQR = 6.1), Internal Factors: *u* = 1,269.50, *p* = 0.006; Risk Factors: *u* = 1,137.0, *p* < 0.001. However, for the External Factor subscale, scores were greater for prison remittals (median = 6, IQR = 1) than for community discharges (median = 5, IQR = 3), *u* = 1,492.50, *p* = 0.001. See Supplementary Table 4 for a breakdown of individual item presence across the two discharge groups.

## Discussion

This paper describes findings from a population of prison-transfer patients detained in MSSs that were discharged into the community or remitted to prison. There were 153 prison-transfer patients discharged from 33 NHS MSSs in England and Wales over a 6 month period, of which, 141 were eligible for inclusion in the above analysis. It was identified that prison-transfer patients detained in MSSs represent a complex subset of patients with a high morbidity of severe mental illness. This cohort was admitted to MSSs on average for less than the recommended stay of 18-months-−2 years (20). Those remitted to prison were more likely to have a primary diagnosis of personality disorder than community discharges, and experienced a considerably shorter length of stay, where over half were admitted for 6 months or less. This short length of stay is concerning considering the apparent vulnerability of this group in regards to their admission histories; for 13 of those remitted to prison, this was their second or subsequent admission to MSSs during their current custodial sentence. These individuals were also more likely to have had a historical admission to MSSs prior to their current custodial sentence and a history of self-harm, including documented self-harm episodes in prison during the period just prior to their admission.

Investigation into clinical presentation prior to discharge was conducted using two validated assessment tools (16, 18). Comparison of the HCR-20v3 total score indicates that those remitted to prison posed an overall higher risk of future violence than did community discharges. The largest difference observed was on the clinical scale; current “dynamic” risk was higher among prison remittals indicating that they had more *recent* problems with psychosocial adjustment in the period preceding discharge. Likewise those remitted to prison were also rated as having more anticipated *future* problems with psychosocial adjustment, based on their goals and plans for the future, as measured by the risk subscale. This is compounded by the presence of fewer protective factors to mitigate risk of future violence within this group. The largest difference observed with the SAPROF was on the motivation subscale; those remitted to prison were rated as having significantly fewer factors on this scale than community discharges, indicating that they had less motivation and lack of positive attitude toward several aspects of treatment. However, these individuals were rated as significantly higher on the external subscale, suggesting the presence of more environmental factors considered beneficial in offering protection from outside of the individual. This likely reflects the nature and the relational security of the environment to which these individuals were remitted. Whilst these individuals may well be considered to be in a contained environment for risk management purposes, their risk of future violence raises issues of public protection regarding the eventual release of these patients back into the community. To add context to these comparative findings, the risk profile for those returned to prison is similar to that observed for people who are discharged into the community from MSS who are observed to be violent at both 6 and 12 months post-discharge (21). In this study we did not follow-up participants who were released into the community post-remittal, however we did observe that 30% of those returned to prison were released during the one-year follow-up period (12). The transition from prison to the community is a vulnerable period, particularly for those with mental health diagnoses, associated with increased risk of adverse outcome (10, 22), therefore it is important that preparation for release includes referral and engagement with community mental health teams and other sources of support post-release (23). As such is was concerning that in our study, less than half of those released into the community within on-year post-remittal were referred to a community mental health team (12).

### The Role of MSS for Persons in Contact With the Criminal Justice System

More people with mental illness are detained in prison in England & Wales than ever before (3). Transfer from prison to MSSs has also increased year-on-year since 2013, representing 66% of admissions to secure psychiatric hospital in 2020 (24). Whilst it is beneficial that more people detained in prison are receiving treatment for their mental health needs, admission of an increasing number of prison-transfers will undoubtedly be putting extra strain on the MSS estate. Admission of prison-transfer patients is just one function of MSSs. These services operate as part of both the wider mental health and criminal justice systems, and have varied roles and responsibilities toward patients depending on their legal status and purpose of admission. For example; admission of patients subject to court directed hospital treatment orders; “step-up/down” of low/high secure patients who require the security conditions of MSSs, and; admission of community patients including those recalled from conditional discharge or community treatment order.

There is wide variation in available resources to deliver care and manage transition pathways for these groups and many different styles of service delivery exist (25). Nevertheless, MSSs are commissioned to provide assessment and/or treatment, and rehabilitation/management of the risk that patients pose to others, with the view to reducing reoffending and the likelihood of relapse (6, 7). This involves undertaking clinical and risk interventions followed by safe discharge of patients to lower levels of security, into the community, or back to prison. As such, it is concerning that many people returned to prison in this study still had significant problems with psychological adjustment at time of prison return and an overall higher risk of future relapse and violence than community discharges. It is clear from these findings that the clinical threshold for appropriate discharge into the community is different to that applied to those returned to prison, indicating that RMOs consider prison as a “safe” discharge destination. However, we evidenced that appropriate and proportionate aftercare is not currently available within the prison estate (12). Likewise, over a third of those returned to prison had a primary diagnosis of Personality Disorder. There are few resources available for people with Personality Disorder in prison, and a significant expansion of services for this patient group is required. Prison mental health services are not able to meet the needs of people returned to prison from MSSs, and the benefits of admission are lost for many upon return to prison (12). For example, we found that, in lieu of appropriate clinical facilities to manage patients' presentation, a fifth of those returned to prison were subsequently placed in segregation. Segregation is not an environment designed or appropriate for those with mental health problems, and its consequences have potential to cause further deterioration (26–28). We also observed that over a fifth of those returned to prison had deteriorated to the point of requiring re-referral to secure psychiatric services. Readmission to in-patient psychiatric services is often used as an indicator of quality of in-patient care and, in this case, may well represent premature discharge from MSSs, especially considering the short admission times observed in this study.

Transition from secure psychiatric care is a time of elevated risk and vulnerability that presents challenges to continuity of care between services, and little good practice guidance exists at present (29, 30). It is our concern that that current guidance does not account for MSSs remittal practices observed in our work, and the difficulties faced by MSSs and prison mental health services when attempting to arrange appropriate aftercare services in the prison estate. As such we have recently secured external funding to conduct a further three-phased mixed-methods investigation, with the objective of gaining a more detailed understanding of current national remittal/aftercare practices. It is the intention to integrate findings from our previous body of work, and our current investigation to improve transparency in remittal practice and to identify core responsibilities of both secure psychiatric services and prison mental health services in order to ensure safe and effective transition back to prison.

There is currently and international drive to modernize forensic mental health services (31–33). The nexus between the criminal justice system and mental health services differs internationally and, at present, there is little work conducted into understanding the remittal care pathway outside of England and Wales. We are aware a recent body of work in Belgium which sought to understand circumstances by which people are returned to prison from secure psychiatric services (13, 14). In this investigation it was observed that all returns to prison were on the basis of the individual subverting security. This practice was observed in our own study, alongside practices such as returning those who are eligible for parole or due to court directed remittal. It is our hope that international colleagues begin to focus their attention on their country's equivalent remittal care pathway and the patients' rights to on-going mental healthcare. We believe that this work would lead to the development and implementation of robust healthcare standards within the international prison estate.

## Strengths and Limitations

This is the first study to describe the characteristics and clinical presentation of a population of prison-transfer patients. This included all prison-transfer patients discharged from all 33 NHS medium secure services over a 6-month period. Interpretation of these findings should be with the caveat that all data were extracted from medical records that may be subject to reporting error or non-reporting. When data items contradicted one another or were unavailable, collateral informants were utilized to strengthen the quality of extracted information (i.e., responsible clinicians and named nurses). Whilst this design allowed for 100% inclusion to be achieved, the study was limited by the fact that patients were not interviewed to aid completion of the validated violence risk assessment tools.

## Data Availability Statement

The raw data supporting the conclusions of this article will be made available by the authors, without undue reservation.

## Ethics Statement

The studies involving human participants were reviewed and approved by North West of England Multi-site Research Ethics Committee (09/H1016/126). Written informed consent for participation was not required for this study in accordance with the national legislation and the institutional requirements.

## Author Contributions

SL drafted the first version of the article. RW, MD, and JS revised the article. All authors accepted the final version of the article.

## Conflict of Interest

The authors declare that the research was conducted in the absence of any commercial or financial relationships that could be construed as a potential conflict of interest.

## Publisher's Note

All claims expressed in this article are solely those of the authors and do not necessarily represent those of their affiliated organizations, or those of the publisher, the editors and the reviewers. Any product that may be evaluated in this article, or claim that may be made by its manufacturer, is not guaranteed or endorsed by the publisher.
