# Supplementary material for: Characteristics of People Returned to Prison From Medium Secure Psychiatric Services in England and Wales: National Cohort Study
Source: Front Psychiatry. 2022 Jun 29;13:881279. doi: 10.3389/fpsyt.2022.881279 (PMC9277066; doi:10.3389/fpsyt.2022.881279)
Supplement: Supplementary file 1 [file Table_1.DOCX]

| Supplementary Table 1. Mental Health Act 1983, Part III:  Sections for patients concerned in criminal proceedings or under sentence | |
| --- | --- |
| Section | Description |
| Remand to Hospital | |
| s. 35 * | Remand to hospital for report on accused person’s mental condition |
| s. 36 * | Remand of accused person to hospital for treatment |
| Hospital and Guardianship Orders | |
| s. 37 | Powers of the courts to order hospital admission or guardianship |
| s. 38 * | Interim hospital order |
| Restriction Orders | |
| s. 41 | Power of higher courts to restrict discharge from hospital  (added to section 37 hospital order) |
| Hospital and Limitation Directions | |
| s. 45A * | Power of higher courts to direct hospital admission (‘hybrid’ order) |
| Transfer to Hospital of Prisoners | |
| s. 47 * | Removal to hospital of persons serving sentences of imprisonment |
| s. 48 * | Removal to hospital of other prisoners |
| s. 49 * | Restriction on discharge of prisoners removed to hospital  (added to sections 47 or 48) |
| *eligible for study inclusion | |

| Supplementary Table 2. Inter-rater reliability for 5 researchers | |
| --- | --- |
| Measure | Reliability score |
| HCR-20: v3 |  |
| Historical | .930 |
| Clinical | .991 |
| Risk management | .994 |
| Total | .989 |
|  |  |
| SAPROF |  |
| Internal | .978 |
| Motivational | .996 |
| External | .889 |
| Total | .988 |

| Supplementary Table 3. Rate Ratio comparisons of HCR 20: V3 individual item presence across prison and community discharges (*n* = 141) | | | | | | | |
| --- | --- | --- | --- | --- | --- | --- | --- |
|  | *n* (%) | | | |  | | |
|  | Total | Community  discharge | Prison  remission | | χ² | *p* | Rate ratio (95% CI) |
|  | (*n* = 141) | (*n* = 49) | (*n* = 92) | |  |  |  |
| **Definitely present (Scored ‘2’)**  Clinical scale (Recent Problems with…) |  |  |  |  | |  |  |
| C1 Insight | 31 (22) | 4 (8) | 27 (29) | 8.37 | | .004 | 3.60 (1.33 - 9.69) |
| C2 Violent ideation or intent | 51 (36) | 7 (14) | 44 (49) | 15.58 | | <.001** | 3.35 (1.63 - 6.87) |
| C3 Symptoms of major mental disorder | 14 (10) | 5 (10) | 9 (10) | .01 | | .936* | 0.96 (0.34 - 2.70) |
| C4 Instability | 34 (24) | 7 (14) | 27 (29) | 3.96 | | .047 | 2.05 (0.97 - 4.37) |
| C5 Treatment or supervision response | 27 (19) | 2 (4) | 25 (27) | 11.01 | | .001** | 6.66 (1.65 - 26.94) |
| Risk Management scale (Future problems with…) |  |  |  |  | |  |  |
| R1 Professional services and plans | 30 (21) | 9 (18) | 21 (23) | .38 | | .538 | 1.24 (0.62 - 2.50) |
| R2 Living situation | 43 (31) | 13 (27) | 30 (33) | .56 | | .455 | 1.23 (0.71 - 2.13) |
| R3 Personal support | 50 (36) | 8 (16) | 42 (46) | 12.01 | | .001** | 2.80 (1.43 - 5.48) |
| R4 Treatment or supervision response | 46 (33) | 9 (18) | 37 (40) | 6.94 | | .008 | 2.19 (1.15 - 4.16) |
| R5 Stress or coping | 72 (51) | 19 (39) | 53 (58) | 4.54 | | .033 | 1.49 (1.00 - 2.21) |
| **Partially/definitely present (Scored ‘1’ or ‘2’)** |  |  |  |  | |  |  |
| Clinical scale (Recent Problems with…) |  |  |  |  | |  |  |
| C1 Insight | 107 (76) | 31 (63) | 76 (83) | 6.54 | | .011 | 1.31 (1.34 - 1.65) |
| C2 Violent ideation or intent | 76 (54) | 16 (33) | 60 (65) | 13.64 | | <.001* | 2.00 (1.30 - 3.07) |
| C3 Symptoms of major mental disorder | 68 (48) | 18 (47) | 50 (54) | 3.97 | | .046 | 1.48 (0.98 - 2.23) |
| C4 Instability | 75 (53) | 15 (31) | 60 (65) | 15.38 | | <.001* | 2.13 (1.36 - 3.33) |
| C5 Treatment or supervision response | 84 (60) | 18 (37) | 66 (72) | 16.27 | | <.001* | 1.95 (1.32 - 2.88 |
| Risk Management scale (Future problems with…) |  |  |  |  | |  |  |
| R1 Professional services and plans | 80 (57) | 21 (43) | 59 (64) | 5.89 | | .015 | 1.50 (1.05 - 2.14) |
| R2 Living situation | 137 (97) | 47 (96) | 90 (98) | .42 | | .516 | 1.02 (1.00 - 1.89) |
| R3 Personal support | 121 (59) | 37 (76) | 84 (91) | 6.55 | | .011 | 1.21 (1.02 - 1.44) |
| R4 Treatment or supervision response | 113 (80) | 34 (69) | 79 (86) | 5.46 | | .020 | 1.24 (1.01 - 1.52) |
| R5 Stress or coping | 137 (97) | 47 (96) | 90 (98) | .42 | | .516 | 1.20 (0.96 - 1.89) |
| * Fishers exact test used as 25% of expected count <5, and/or observed counts < 1  * * Also significant at the Bonferroni corrected alpha, p < .0025 | | | | | | | |
|  | | | | | | | |

| Supplementary Table 4 . Rate Ratio comparisons of SAPROF individual item presence across prison and community discharges (*n* = 141) | | | | | | | |
| --- | --- | --- | --- | --- | --- | --- | --- |
|  | | *n* (%) | | |  |  |  |
|  | | Total | Community  discharge | Prison  remission | χ² | *p* | Rate ratio (95% CI) |
|  | | (*n* = 141) | (*n* = 49) | (*n* = 92) |  |  |  |
| **Definitely present (Scored ‘2’)** | |  |  |  |  |  |  |
| Internal items | |  |  |  |  |  |  |
| 2 Secure attachment in childhood | | 59 (43) a | 26 (55) | 33 (37) | 4.38 | .036 | 0.66 (0.46 - 0.96) |
| 4 Effectively coping strategies | | 28 (20) b | 18 (38) | 10 (11) | 13.48 | <.001** | 0.30 (0.15 - 0.59) |
| 5 Self-control | | 61 (43) | 33 (67) | 28 (30) | 17.75 | <.001** | 0.45 (0.31 - 0.65) |
|  | |  |  |  |  |  |  |
| Motivational item | |  |  |  |  |  |  |
| 6 Stable work situation | | 3 (2) | 3 (6) | - | 5.76 | .040* | - |
| 7 Structured leisure activities | | 57 (40) | 27 (55) | 30 (33) | 6.72 | .010 | 0.59 (0.40 - 0.87) |
| 9 Motivation for treatment | | 47 (33) | 25 (51) | 22 (24) | 10.57 | .001** | 0.47 (0.30 - 0.74) |
| 10 Positive attitude towards authority | | 48 (35) c | 27 (57) | 21 (23) | 16.49 | <.001** | 0.40 (0.25 - 0.62) |
| 11 Positive life goals | | 34 (26) d | 24 (51) | 10 (12) | 24.84 | <.001** | 0.23 (0.12 - 0.44) |
| 12 Motivated to use medication effectively | | 59 (47) e | 28 (61) | 31 (39) | 5.46 | .020 | 0.65 (0.45 - 0.92) |
|  | |  |  |  |  |  |  |
| External items | |  |  |  |  |  |  |
| 13 Social network | | 24 (17) | 12 (25) | 12 (13) | 2.97 | .085 | 0.53 (0.26 - 1.10) |
| 14 Intimate relationship | | 13 (9) | 8 (16) | 5 (5) | 4.53 | .037* | 0.33 (0.12 - 0.96) |
| 15 Professional care | | 21 (15) | 19 (39) | 2 (2) | 33.79 | <.001** | 0.06 (0.01 - 0.23) |
| 16 Living circumstances | | 100 (68) | 12 (25) | 88 (96) | 78.51 | <.001** | 3.91 (2.38 - 6.40) |
| 17 External control | | 96 (69) f | 6 (13) | 90 (98) | 106.56 | <.001* | 7.83 (3.70 - 16.55) |
| **Partially/definitely present (Scored ‘1’ or ‘2’)** | | | | | | | |
| Internal items | |  |  |  |  |  |  |
| 2 Secure attachment in childhood | | 92 (67) a | 34 (72) | 58 (64) | 0.87 | .350 | 0.89 (0.71 - 1.13) |
| 4 Effectively coping strategies | | 92 (62) b | 40 (83) | 52 (58) | 9.20 | .002** | 0.69 (0.56 - 0.86) |
| 5 Self-control | | 118 (84) | 46 (94) | 72 (78) | 5.71 | .017 | 0.83 (0.73 - 0.95) |
|  | |  |  |  |  |  |  |
| Motivational item | |  |  |  |  |  |  |
| 6 Stable work situation | | 23 (16) | 17 (35) | 6 (7) | 18.59 | <.001** | 0.19 (0.08 - 0.45) |
| 7 Structured leisure activities | | 114 (81) | 43 (88) | 71 (77) | 2.31 | .138 | 0.88 (0.76 - 1.02) |
| 9 Motivation for treatment | | 95 (67) | 40 (82) | 55 (60) | 6.94 | .008 | 0.73 (0.59 - 0.91) |
| 10 Positive attitude towards authority | | 99 (91) c | 43 (92) | 56 (61) | 21.81 | <.001** | 0.62 (0.53 - 0.74) |
| 11 Positive life goals | | 72 (54) d | 37 (79) | 35 (41) | 17.70 | <.001** | 0.52 (0.39 - 0.70) |
| 12 Motivated to use medication effectively | | 104 (83) e | 46 (100) | 58 (73) | 14.70 | <.001** | 0.73 (0.62 - 0.84) |
|  | |  |  |  |  |  |  |
| External items | |  |  |  |  |  |  |
| 13 Social network | | 102 (72) | 42 (86) | 60 (65) | 6.71 | .010 | 0.76 (0.63 - 0.92) |
| 14 Intimate relationship | | 23 (16) | 13 (27) | 10 (11) | 5.74 | .017 | 0.41 (0.19 - 0.87) |
| 15 Professional care | | 131 (93) | 46 (94) | 85 (92) | 0.11 | .743 | 0.98 (0.90 - 1.08) |
| 16 Living circumstances | | 132 (94) | 41 (84) | 91 (99) | 12.43 | <.001** | 1.18 (1.04 - 1.34) |
| 17 External control | | 128 (91) f | 36 (74) | 92 (100) | 25.16 | <.001** | 1.33 (1.32 - 1.57) |
| a community *n* = 47, prison *n* = 90  b community *n* = 48, prison *n* = 90  c community *n* = 47, prison *n* = 92  d community *n* = 47, prison *n* = 86  e community *n* = 46, prison *n* = 79  f community *n* = 48, prison *n* = 92 | * Fishers exact test used as 25% of expected count <5, and/or observed counts < 1  * * Also significant at Bonferroni corrected alpha, p < .0033 | | | | | | |
